# Generation of a High-Affinity Nanobody Against CD147 for Tumor Targeting and Therapeutic Efficacy Through Conjugating Doxorubicin

**DOI:** 10.3389/fimmu.2022.852700

**Published:** 2022-05-04

**Authors:** Rifei Li, Xinjie Zhu, Peng Zhou, Yuehua Qiao, Yinqian Li, Yice Xu, Xi Shi

**Affiliations:** ^1^ College of Veterinary Medicine, Northwest A&F University, Yangling, China; ^2^ Key Laboratory of Tropical Biological Resources of Ministry of Education, School of Pharmaceutical Sciences, Hainan University, Haikou, China; ^3^ Synthetic and Functional Biomolecules Center, College of Chemistry and Molecular Engineering, Peking University, Beijing, China; ^4^ Artificial Auditory Laboratory of Jiangsu Province, Xuzhou Medical University, Xuzhou, China; ^5^ Department of Otolaryngology-Head and Neck Surgery, Xiaogan Hospital Affiliated to Wuhan University of Science and Technology, Xiaogan, China

**Keywords:** tumor targeting, nanobody, CD147, phage display, DOX–11-1

## Abstract

CD147, a glycosylated transmembrane protein in the immunoglobulin superfamily, is overexpressed on the surfaces of various tumor cells and promotes cancer cell proliferation, invasion, and metastasis. Nanobodies, characterized by small sizes, high affinities and specificities, and low immunogenicities, are promising diagnostic and therapeutic tools. However, there are few reports on nanobodies that specifically target CD147. In this work, a specific anti-CD147 nanobody has been successfully identified using phage display technology. The tumor target and antitumor effects have also been detected in different CD147-positive tumors in *in vitro* and *in vivo* assays, respectively. Meanwhile, it has a synergistic effect for inhibiting 4T1-bearing mice through conjugating doxorubicin. It may afford new strategies for cancer therapies.

## Introduction

CD147, also known as extracellular matrix metalloproteinase inducer (EMMPRIN), a member of the immunoglobulin (Ig) superfamily, is a type I transmembrane glycoprotein (1, 2). It is composed of N-terminal signal peptides, an extracellular portion including two Ig domains, a transmembrane region with binding activity, and a short cytoplasmic region with 39 amino acid residues. The CD147 protein has two forms with low and high degrees of glycosylation [LG-CD147 (~36 kDa) and HG-CD147 (~40–60 kDa)] (3), which are related to its functions. Jia et al. (4) demonstrated that the HG-CD147 molecule plays crucial roles in tumor cell invasion and metastasis, whereas the LG-CD147 molecule showed reduced adhesion capability *in vitro* because it lacked the ability to induce matrix metalloproteinase (MMP) expression.

Additionally, CD147 is expressed widely in a few normal tissues and in most pathological tissues (5, 6); it is especially highly expressed in a variety of malignant tumors (7, 8). It can facilitate MMP secretion from tumor cells, fibroblasts, and endometrial cells, which can lead to the destruction of the extracellular matrix and basement membrane. This effect significantly promotes tumor cell proliferation, invasion, and metastasis (9–11), suppressing cell apoptosis and anoikis (12, 13). CD147/EMMPRIN has been shown to affect chemotherapy efficacy (14–16), neovascularization (17), and radiation resistance. Zhu et al. (18) reported that CD147 overexpression in ovarian carcinoma tissues facilitated the drug resistance of human ovarian cancer cells. CD147 has been found to be associated closely with human malignancies such as liver, lung, breast, colorectal, prostate, and bladder cancers, as well as glioma and salivary duct carcinoma (19–22). Its expression in metastatic breast cancer proves that it correlated with monocarboxylate transporters (MCTs), which are associated with lactate efflux transport (23), and it showed that CD147 can regulate MCT stability, function, and expression on cell surfaces (24). CD147 is regarded as a tumor-related biomarker that is important for disease diagnosis, prognostic assessment, and targeting therapy (25). Therefore, the development of CD147 antibodies is particularly urgent due to its broad application prospects for cancer therapies.

Antibody-mediated targeting therapy, which serves as a new cancer therapeutic strategy, has become an especially hot research area (26–28). Among them, the anti-CD147 monoclonal antibody HAb18 was generated by hepatocellular carcinoma suspension cells. The ^131^I-labeled HAb18G/CD147-specific monoclonal antibody F(ab’)2 has been used to treat liver cancer and has shown good clinical efficacy (29, 30). Moreover, the monoclonal antibodies are extracted principally from mouse hybridomas in that they have high affinities and can specifically recognize relative antigens (31). They have been widely applied in scientific studies and clinics, but they have some limitations more or less. For instance, they have large molecular weights that may limit tumor penetration and affect their tissue distribution (32, 33). Additionally, the traditional monoclonal antibody may cause adverse events because it is off-target (34). Hamers-Casterman et al. (35) reported the natural occurrence of a heavy-chain antibody (containing only heavy chains and no light chain) in Camelidae (e.g., dromedary, Bactrian camel, and llama), which has been designated a nanobody (Nb) or the variable domain of the heavy chain of a heavy-chain antibody (VHH) and has an average size of 15 kDa. Nbs have high affinity, stability, aqueous solubility, and expression level and low immunogenicity (36). These properties arise from their single-domain nature and pivotal amino acid mutations in the framework 2 region, which result in a more hydrophilic overall structure compared with conventional antibody fragments. The complementarity-determining region (CDR3) loops of VHHs are extended, which increases affinity by further enabling the recognition of cavities and hidden epitopes on antigen surfaces. Moreover, Nbs exhibit low immunogenicity due to their high-homology to human VH sequences (37). Based on these properties, several research groups have developed currently exploitable Nbs against tumor-specific receptors as drug delivery carriers to tumors, which reduces drug toxicities to normal cells (38). Recently, anti-epidermal growth factor receptor (EGFR) Nb was conjugated on the surfaces of liposomes to downregulate the EGFR level (39). Meanwhile, anti-human epidermal growth factor receptor 2 (HER2) Nb has been proven to be useful for the optical molecular imaging of HER2-positive breast cancer *in vitro* and *in vivo* (40). Thus, Nbs are new tools for disease diagnosis and therapies.

**Figure 1:**
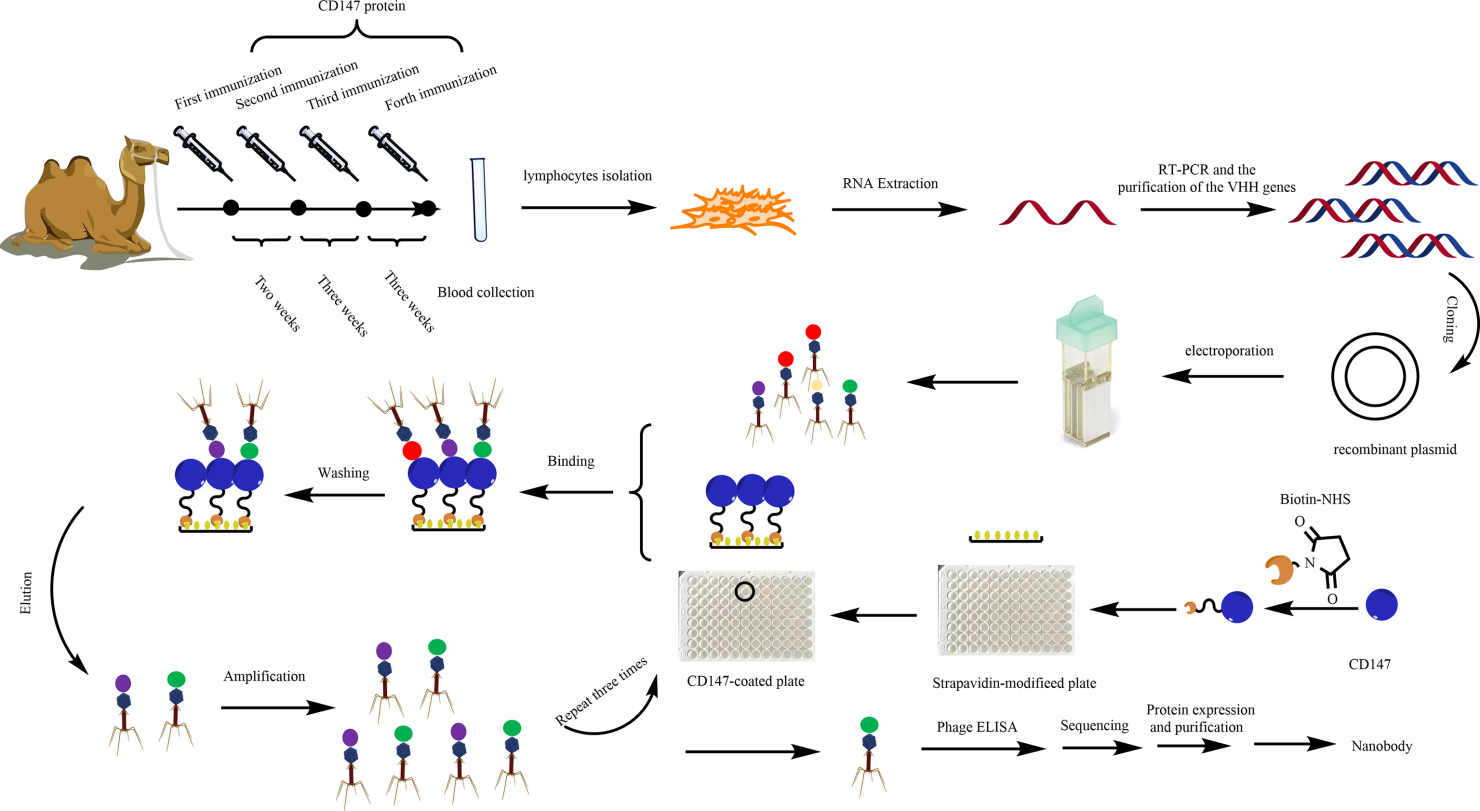
Diagram of the strategies for anti-CD147-VHH library construction and the screening, expression, and purification of anti-CD147 Nb.

However, there are few reports on the Nbs that specifically target CD147. In this study, we constructed a VHH library by immunizing a camel with CD147 protein (**Figure 1**). We screened CD147 Nbs *via* phage display technology. Among them, 11-1, which served as one of the anti-CD147 Nb candidates, was expressed by *Escherichia coli*. We performed a series of verification assays to confirm the specificity and affinity of 11-1. Meanwhile, we conjugated Nb 11-1 with doxorubicin (DOX) *via* a chemical linker and found that DOX-11-1 has good antitumor efficacy for the 4T1 breast tumor model in Balb/c mice.

## Materials and Methods

### Construction of Anti-CD147-Specific Library

A healthy alpaca was immunized through five subcutaneous neck injections of the ecto-domain protein of CD147 (**Figure S1**) (300 μg, 2 ml of protein and 2 ml of adjuvant, kindly provided by Professor Bin Xia of Peking University; Sigma) (41) blended with the same volume of Freund’s complete adjuvant (Sigma) the first time and Freund’s incomplete adjuvant the next four times. After the fifth immunization, 30 ml of blood containing anti-coagulant was collected and diluted with an equal volume of sterile phosphate-buffered saline (PBS). Total RNA was isolated from 10^8^ peripheral blood lymphocytes and reverse transcribed using Invitrogen Superscript III reverse transcriptase to synthesize complementary DNA (cDNA). To obtain the VHH gene, we used polymerase chain reaction (PCR) to amplify the gene of interest (42). The first PCR was performed using the first-strand cDNA as a template with the primers AlpVh-LD (5’-CTTGGTGGTCCTGGCTGC-3’) and CH2-R (5’-GGTACGTGCTGTTGAACTGTTCC-3’). The protocol mainly contains an initial denaturation step at 94°C for 7 min, followed by 30 cycles of 94°C for 1 min, 55°C for 1 min, and 72°C for 1 min, and a final extension step at 72°C for 10 min. The PCR products were purified by agarose gel electrophoresis and used as the template for the second PCR.

The framework-1 (AlpVh-*Sfi*I: 5’-TCGCGGCCCAGCCGGCCATGGCCCAGKTGCAGVCTCGTGGAGTCNGGNGG-3’ and AlpVHHR1-*Not*I: 5’-CGAGTGCGGCVCGCGGGGTCTTCGCTGTGGTGCG-3’) and framework-2 (AlpVh-*Sfi*I: 5’-TCGCGGCCCAGCCGGCCATGGCCCAGKTGCAGCTCGTGGAGTCNGGNGG-3’ and AlpVHHR2-*Not*I: 5’-CGAGTGCGGCCGCTTGTGGTTTTGGTGTCTTGGG-3’) primers were used to amplify the Nb repertoire, and the final products were extracted by agarose gel purification. The purified final PCR fragments (single variable domain on a heavy chain, VHH) were ligated into the phagemid vector pHEN1 at 16°C for 16 h using T4 DNA ligase (NEB, USA), after digesting with the restriction enzymes *Sfi*I and *Not*I (NEB, USA). The ligation products were electro-transformed into fresh *E. coli* TG1 competent cells. After helper phage M13KO7 (Amersham Biosciences Corp) was added, phages were precipitated *via* the PEG-NaCl solution for the phage display CD147 Nb library.

### The Panning of Anti-CD147 Nanobody

To obtain specific VHH antibodies, the synthetic VHH library further screened Nbs against CD147. Briefly, the Maxisorp 96-well plates were coated with 100 μl of 50 μg/ml CD147 protein/well at 4°C overnight. Another well with 100 μl of PBS was used as a non-coated control. Wells were washed three times with PBST [PBS, supplemented with 0.05% (v/v) Tween-20] and blocked with 200 μl of 4% BSA in PBS at 37°C for 2 h. After three washes with PBST, 100 μl of Nb display phage was added into each well at 37°C for 1 h with gentle shaking, followed by washing five times with PBST and adding 100 μl of 0.1 M Glycine-HCl (pH 2.2), and then incubated for 8 min to elute specific binding phages. *E. coli* TG1 cells were infected with eluted phages at 37°C for 30 min; the part of the infected *E. coli* TG1 cells grown on LB agar was supplemented with 100 μg/ml ampicillin. The phage titers were calculated through the number of colonies. With the help of helper phages (M13KO7), the others were used for next panning. After three rounds of panning, we screened 70 colonies to further identify positive colonies.

### Identification of CD147-Specific VHH Antibodies With Indirect Phage-ELISA

ELISA was conducted to panning for CD147-binding VHH. The procedure was described as in a previous report (43); single colonies of phage-infected cells were grown in 1 ml of LB culture medium supplemented with 100 μg/ml ampicillin at 37°C and 220 rpm for 5 h and then 100 μl of cells was picked from another fresh LB culture medium (1 ml) supplemented with 100 μg/ml ampicillin and incubated at 37°C and 220 rpm until OD_600_ reached ~0.6. M13KO7 helper phages (10^8^) were added, followed by incubation for 1 h at 37°C. The cells were pelleted at 5,000 rpm for 10 min, and the supernatants were replaced with fresh LB medium (1 ml).

Maxisorp 96-well plates were coated with 100 μl of 50 μg/ml CD147 protein/well at 4°C overnight. Wells were washed three times with PBST [PBS, supplemented with 0.05% (v/v) Tween-20] and blocked with 200 μl of 4% BSA in PBS at 37°C for 2 h. After three washings with PBST, the treatment group was added resuspended phages while the control group was added 10^8^ M13KO7 helper phages then incubated for 1 h at 37°C. Plates were washed and then incubated with horseradish peroxidase (HRP)-conjugated goat anti-M13 specific antibody for 1 h at room temperature. Plates were washed and then developed by adding 100 μl of tetramethylbenzidine (TMB) for 5 min, then the reaction was stopped by adding 50 μl of 2 M H_2_SO_4_. Absorbance at 450 nm was measured with a microplate reader. The absorbance of the treatment group as triple or above that of the control group served as positive colonies that were sent for sequencing followed by multiple sequence alignment analysis, performed using DNAMAN software.

### Expression and Purification of CD147 Nanobodies

The gene of interest containing a positive phage Nb was cut and digested by the restriction enzymes *Nde*I and *Sal*I (NEB). Then, the gene fragment was gel-purified and ligated into a pET-15b(+) vector (preserved in our laboratory). The ligation products were transformed into fresh *E. coli* BL21(DE3) competent cells (Jinsirui Biotech) and cultured in LB medium without ampicillin at 37°C with shaking at 220 rpm for 1 h. They were then coated on LB/ampicillin agar [containing 100 μg/ml ampicillin and 20% (w/v) agar] plates and incubated at 37°C overnight. The next day, single colonies were selected randomly, cultured in LB medium supplemented with 100 μg/ml ampicillin, and incubated at 37°C with shaking at 220 rpm for 4 h. When OD_600_ reached 0.6–0.8, the VHH antibody colonies were induced with 1 mM (w/v) isopropylthio-β-D-galactoside (IPTG) and incubated at 16°C with shaking at 140 rpm for 14 h. The cells were centrifuged and broken ultrasonically to obtain cellular extract. The CD147-specific Nbs were purified on ÄKTA start (GE Healthcare) with a His-Trap™ HP column by sequential eluting with 10, 50, and 500 mM imidazole solutions. The size and purity of the VHH antibodies were examined by sodium dodecyl sulfate polyacrylamide gel electrophoresis (SDS-PAGE). Then, the Nb proteins were concentrated using an ultrafiltration column and dialyzed into PBS (pH 7.4) for further study. Additionally, we constructed EGFP-11-1, which contained the EGFP domain and the 11-1 domain. Among them, the EGFP domain was fused into the C terminus of 11-1 through a GGGGS linker. Its expression and purification protocols were the same as those of 11-1.

### Characterization of 11-1 and EGFP-11-1

The particle sizes of Nb 11-1 and EGFP-11-1 were measured by dynamic light scattering (DLS) using a Zetasizer Nano ZS90 device (Malvern Instruments, Malvern, UK). The Nb 11-1 (1 mg/ml) morphology was observed by transmission electron microscopy (TEM).

### Cell Culture

HeLa (44), 4T1 (45), U87 (46), and 293T (low expression of CD147) cells were preserved in our laboratory (purchased from ATCC), and the human hepatic carcinoma cell line SMMC-7721 (wild-type) (47), which has been authenticated by the STR profile, was kindly provided by Professor Bin Xia (Beijing Nuclear Magnetic Resonance Center, School of Life Sciences, and College of Chemistry and Molecular Engineering, Peking University). HeLa, U87, and 293T cells were maintained in DMEM (GE Healthcare) supplemented with 10% fetal bovine serum (FBS) and 100 U ml^−1^ of penicillin–streptomycin. 4T1 cells were maintained in RP1640 (GE Healthcare) supplemented with 10% FBS and 100 U ml^−1^ of penicillin–streptomycin. All cells were grown in a 5% CO_2_ humidified atmosphere at 37°C.

### The Analysis of Binding Affinity Between CD147 and Nanobodies

The binding affinities of CD147 and C78 to Nbs were individually measured by BLI analysis using the ForteBio Octet system. Briefly, 0.18 mg of Sulfo-NHS-LC-Biotin (Pierce) was dissolved in 100 µl of ultrapure water and added to the protein solutions separately (700 μg for CD147 and 445 μg for C78), followed by incubation of the reaction in a freezer for 2.5 h. A desalting column was equilibrated by adding 2.5 ml of PBS to the top of the resin bed and centrifuging at 1,000 × *g* for 2 min. This step was repeated two or three times. The biotinylated proteins were purified by centrifugation of the desalting column at 1,000 × *g* for 2 min.

Interactions of Nb 11-1 with CD147 and C78 were detected by BLI in four steps. During the equilibration step, the sensors were immersed in PBST for 60 s. Biotin-labeled CD147 and C78 were immobilized on top of the sensors by immersing the sensors in 200 μl of the biotin-labeled protein solutions at room temperature for 200 s. Nb 11-1 was then diluted in different concentrations of PBST, and the sensors were immersed in the Nb 11-1 solutions for 200 s. Finally, the sensors were immersed in PBST for 100 s to remove the free Nb 11-1. All steps were performed in a 200-μl volume at room temperature with shaking at 1,000 rpm.

### Immunoblot Analysis

To determine whether the screened Nb can bind specifically to the epitope of the CD147 protein, HRP (Xingbao Biotech) was coupled to the Nbs. Briefly, 12 μl of REAGENTIA activated horseradish oxidase was mixed with 40 μg of CD147 Nbs solution. Next, 10 μl of REAGENTII was added to adjust the pH to approximately 9.5 and incubated for 30 min at 37°C. Then, 30 μl of REAGENTIII was added to ensure that the pH of the enzyme conjugate was approximately 7.0. After that, a small amount of NaBH_4_ was mixed and glycerol was added. Finally, the mixture was stored at −20°C. The equivalent of related proteins [CD147 and C78 proteins (the C-terminal domain protein structure of CD147)] was measured by Western blot analysis. The protein concentration of the CD147 or C78 and cell lysates was measured using a Protein Analyzer (Pultton). Protein (500 ng) was fractionated on a 12% SDS-PAGE gel and transferred to polyvinylidene fluoride (PVDF) membranes by electrophoresis. Membranes were blocked for 2 h at room temperature using 5% skimmed milk followed by a 1-h incubation with HRP-conjugated VHH antibodies. Reactive bands were visualized with Immobilon™ Western Chemiluminescent HRP Substrate (Millipore).

### Cell Fluorescence Analysis

Cells were cultured in confocal microscope dishes for 24 h in a 5% CO_2_ humidified atmosphere at 37°C. The next day, cells were treated with EGFP and equivalent EGFP-11-1 on ice for 15 min, respectively. Subsequently, the cell culture medium was removed, and the cells were washed gently three times with PBS. Cell fluorescence was detected under a confocal microscope *via* 488 nm laser (Nikon, Tokyo, Japan).

### *In Vivo* Tumor Imaging

Eight 5- to 6-week-old female Balb/c mice were implanted with 4T1 tumor cells in the right leg *via* subcutaneous injection. *In vivo* fluorescence imaging was performed using an imaging system. A filter (625/700 nm excitation/emission) was used to obtain fluorescence. The Balb/c mice were injected intravenously with EGFP-11-1-Cy5.5 (7.5 μM) or the same dose of EGFP-Cy5.5 *via* the tail vein. PBS-injected mice were used as blank controls. The mice were anesthetized with isoflurane furane, and images were acquired at the indicated time points in **Figure 4**.

### Synthesis of DOX–11-1

DOX-maleimide was synthesized as described previously (48). Briefly, 66.4 mg (14.5 μmol) of DOX was dissolved in 10 ml of dimethyl sulfoxide (DMSO), 30 μl TEA was added, and the mixture was stirred for 30 min, and then 33.5 mg (126 μmol) SMP was added. The reaction was stirred at 37°C and 220 rpm for 48 h. The disappearance point of the starting material was monitored by thin-layer chromatography. The product was purified by high-performance liquid chromatography (HPLC) and identified by LC mass spectrometry (LC/MS).

DOX–11-1 was synthesized. Briefly, 800 μl of 1× PBS (1 mmol/L EDTA, pH 9.0) was mixed with 200 μl of 0.35 mM 11-1 and 200 μl of 11 mM DOX-maleimide at 25°C overnight. The free DOX-maleimide was removed using a desalting column to obtain the DOX–11-1 product.

### The Cellular Distribution of DOX–11-1

The 293T (negative control), U87, HeLa, and 4T1 cell lines were seeded in laser confocal plates at a density of 1 × 10^4^ cells/well. The next day, DOX–11-1 and free DOX were added at concentrations of 5 and 2.5 μM, respectively. Following 12- and 24-h interventions with DOX–11-1 and free DOX, the cell culture medium was removed, the cells were washed gently three times with PBS, and the fluorescent signal of DOX was observed by confocal laser scanning microscopy.

### Cell Proliferation Assay

To assess the inhibitory effects of 11-1, free DOX, and DOX–11-1 on 293T, U87, and 4T1 cells, MTT assays were performed. The cells were seeded into 96-well cell culture plates and incubated at 37°C overnight. The next day, different concentrations (0, 0.625, 1.25, 2.5, and 5 μM) of drugs (11-1, free DOX, and DOX–11-1) were mixed with the tumor cells, followed by incubation for 24 h. A 10-μl volume of MTT (10 mg/ml) was added to each well, and the samples were incubated again for 4 h. The supernatant was discarded, an aliquot of 150 μl DMSO was added to each well, and the crystals were fully dissolved by shaking. Absorbance at 490 nm was measured in a microplate reader. The cell proliferation inhibition rate (%) was calculated using the equation (1 − OD_sample_/OD_control_) × 100.

### Cell Apoptosis Assay

Cells were seeded in 12-well plates and incubated with various concentrations (5 μM) of 11-1, DOX, and DOX–11-1 for 24 h. Cell apoptosis was investigated using an annexin V-FITC apoptosis detection kit (Becton Dickinson). The cells were double-strained using annexin V-FITC and propidium iodide (PI), and apoptosis rate was investigated by flow cytometry.

### *In Vivo* Antitumor Study

Twenty 5- to 6-week-old female Balb/c mice were injected subcutaneously with 3 × 10^6^ 4T1 cells suspended in 100 μl of PBS. When the average tumor volume reached approximately 200 mm^3^, the mice were divided randomly into four groups treated with DOX (0.1 mg equivalent/kg), 11-1, DOX–11-1 (equivalent to 0.1 mg DOX equivalent/kg), and 0.9% NaCl, respectively. The interval time for drug treatments was 36 h. The tumor volume and body weight were measured every 2 days. The tumor volume was calculated using the equation 1/2 × length × width^2^. All tumors were divided on day 16 after the administration of excessive anesthesia. The toxicity of the drugs was evaluated by measuring body weight changes in the mice.

### Statistical Analysis

Statistical analysis was performed by one- or two-tailed Student’s *t*-tests, and the results were shown as mean ± standard error of mean (SEM). Significance threshold (*p*-value) was shown in the corresponding figure captions. *p < 0.05, **p < 0.01, ***p < 0.001, ****p < 0.0001.

## Results

### Immunized VHH Library Construction and Nb Screening

After the fifth immunization, the titer of the camel’s serum was determined, by checkerboard titration, to be approximately 1:128,000 (data not shown), indicating that CD147 immunization had been completed as needed for library construction and screening (**Figure 1**). The total size of the phage display VHH library was approximately 5 × 10^8^ colony-forming units, meaning that it matched the diversity and specificity standards of the VHH antibody library, and could be used for subsequent screening.

**Figure 2:**
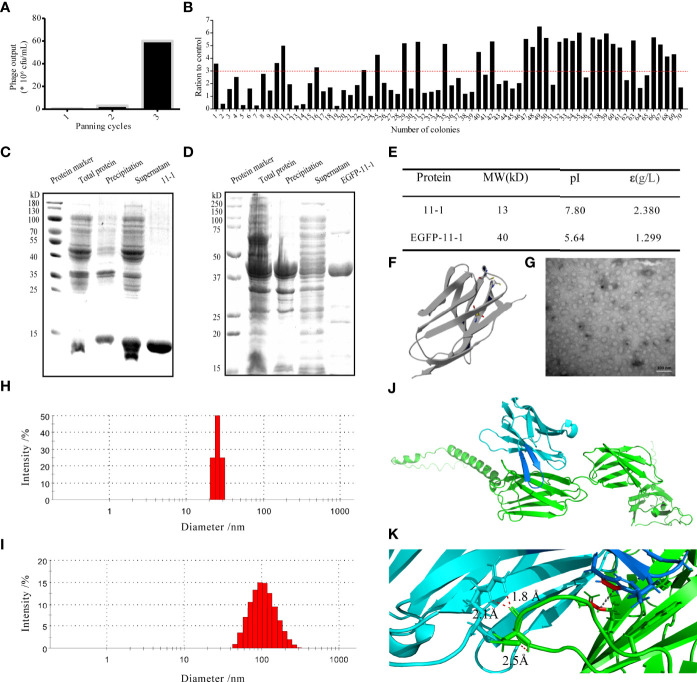
The characterization of the candidate anti-CD147 Nb 11-1. **(A)** Enrichment over three rounds of panning. The histogram shows the enriched quantities and the enriched fold changes after each round. **(B)** Phage ELISA for the selection of 70 colonies from the three rounds. Positivity was defined by positive/negative ratios >3. **(C, D)** Analysis of Nb 11-1 **(C)** and EGFP-11-1 **(D)** expression and purification by SDS–PAGE. **(E)** Properties of Nb 11-1 and EGFP–11-1, determined using the ExPASy ProtParam tool. **(F)** The predicted spatial structure of Nb 11-1. **(G)** The TEM image of Nb 11-1 (scale bar = 100 nm). **(H, I)** The particle size distribution of Nb 11-1 **(H)** and EGFP-11-1 **(I)**. **(J)** The docking analysis between Nb 11-1 (cyan and blue) and CD147 (green). **(K)** The enlarged image from J to show the involving amino acids of Nb 11-1 in the interaction with CD147 *via* AlphaFold2 software.

We screened anti-CD147 Nbs *via* phage display technology (49). The positive phage clones against CD147-specific VHH were enriched efficiently after three rounds of panning. Compared with the first screening round, 120-fold (Output in Round 3/Round 1 = 60/0.5) greater specific Nb enrichment was observed after the third round (**Figure 2A**). To obtain CD147-specific VHH with good diversity and high affinity, 70 randomly selected colonies were examined by phage ELISA. Among them, 29 clones were positive (**Figure 2B**) (positivity was defined as a positive/negative ratio > 3). The 29 positive clones were sequenced and analyzed using the DNAMAN sequence alignment software (**Figure S2**). Taken together, the 11-1 was selected as a candidate clone for subsequent studies due to expression yield and specificities (**Figure S3**).

### Purification and Characterization of 11-1 and EGFP-11-1

To obtain the VHH Nb 11-1 and the EGFP-VHH fusion protein EGFP-11-1, the coding genes for 11-1 and EGFP-11-1 were cloned into the pET-15b and pET-25b vectors, respectively. The nucleobase and amino acid sequences of the 11-1 Nb are shown in **Figure S4**. These expression vectors were transformed into *E. coli* BL21(DE3) cells and carried a His-tag to facilitate purification by ÄKTA start with a His-Trap™ HP column. Anti-CD147 Nb (11–1) and the EGFP-VHH fusion protein (EGFP-11-1) were purified on the His-Trap™ HP column using 250 mM imidazole for elution. The identification of purified proteins and measurement of their molecular weights and purities were carried out by SDS-PAGE. It indicated that the molecular weight of 11-1 and EGFP-11-1 fusion protein was approximately 13 and 40 kDa, respectively (**Figures 2C, D**). The final yield of the expressed Nb after IPTG induction in 1 L of bacterial liquid was approximately 18.9 mg (data not shown). The molecular weights, isoelectric points, extinction coefficients, instability indices, and aliphatic indices of the purified proteins were calculated using ExPASy and are shown in **Figure 2E**. The predicted spatial structure of 11-1 is shown in **Figure 2F** *via* Phyre2 online software (http://www.sbg.bio.ic.ac.uk/ph-yre). The size and morphology of 11-1 were evaluated using TEM (**Figure 2G**). DLS showed that 11-1 and EGFP–11-1 had particle sizes of approximately 27 and 100 nm, respectively (**Figures 2H, I**). Because the molecular weight of protein was dependent on its amino acid sequences. As shown in **Figures 2C–E**, the predicted molecular weights of 11-1 and EGFP-11-1 were consistent with SDS-PAGE results. However, the diameter of the protein was dependent on the protein’s complex space structures, protein folding, and so on. It implied that the diameter and molecular weight did not have a direct matching relationship. Meanwhile, we analyzed interaction between Nb 11-1 and CD147 *via* AlphaFold2 software (50); it indicated that W98 in the CDR3 domain of Nb 11-1 forms a hydrophobic core with R300 of the CD147 β-sheet, and R45 upstream of CDR3 forms a strong double hydrogen bond with D260 of CD147 to assist antibody binding. In addition, N102 of Nb 11-1 may form hydrogen bonds with the phenolic hydroxyl group of Y256 of CD147 to further stabilize binding, thus obtaining an antigen–antibody complex with a high binding force (**Figures 2J, K**).

### 11-1 Nanobody Had High Affinity for CD147

**Figure 3:**
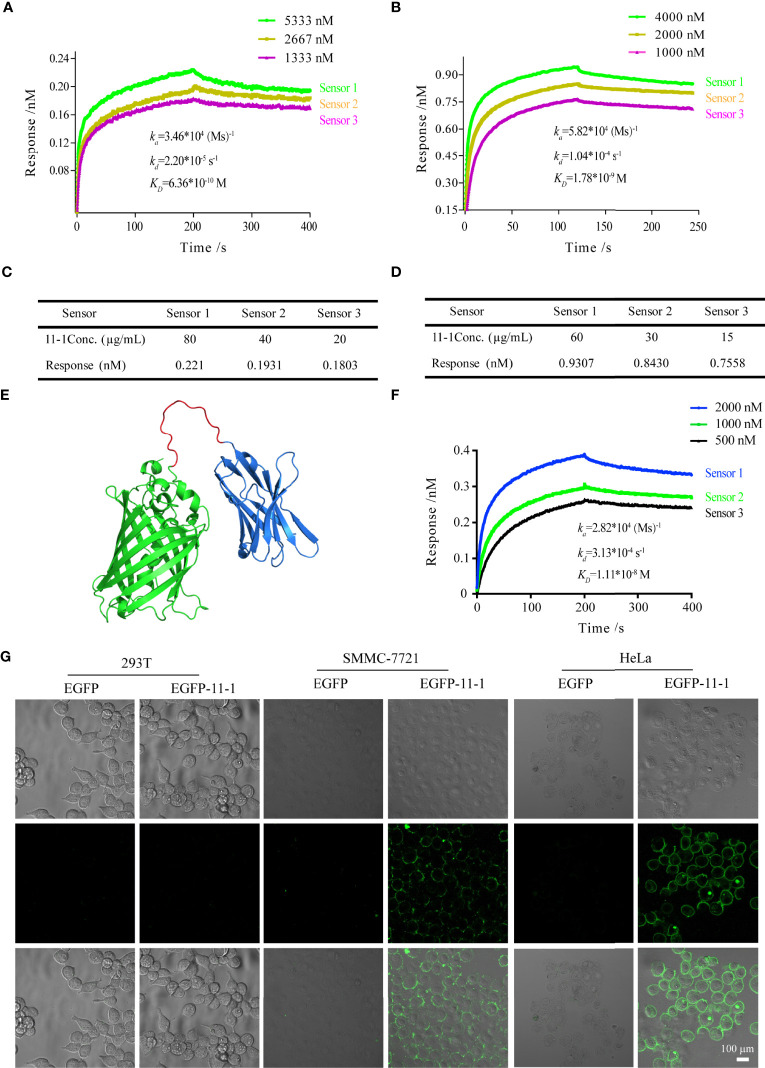
The tumor-targeting effects of anti-CD147 nanobody *in vitro* assays. **(A)** The interaction of kinetics between 11-1 and CD147. **(B)** The interaction of kinetics between 11-1 and C78. **(C)** The response of CD147 corrected with 11-1 concentration. **(D)** The response of C78 corrected with 11-1 concentration. **(E)** The predicted spatial structure of EGFP-11-1. **(F)** The interaction of kinetics between CD147 and EGFP-11-1. **(G)** Cell fluorescence analysis assay. The cell lines involved 293T, SMMC-7721, and HeLa; EGFP-11-1 (2 mg/ml, 20 μl) and EGFP (2 mg/ml, 20 μl) were incubated with these cells at 4°C for 15 min, and these cells were washed three times with PBS. 293T cells and EGFP protein were used as negative controls. Scale bar = 100 μm.

We performed BLI analysis with ForteBio Octet to confirm the affinity of Nb 11-1 for CD147 and C78 (C-terminal ecto-domain protein of CD147). The kinetic parameters of Nb 11-1 interactions with CD147 and C78 were determined, respectively. The association and dissociation rates of Nb 11-1–CD147 were 3.46 × 10^4^ M s^−1^ and 2.20 × 10^−5^ s^−1^, respectively. The corresponding values for C78 were 5.82 × 10^4^ M s^−1^ and 1.04 × 10^−4^ s^−1^. Equilibrium dissociation constants (which represent the affinity of the interaction between two molecules) for Nb 11-1 interaction with CD147 and C78 were 6.36 × 10^−10^ M and 1.78 × 10^−9^ M, respectively (**Figures 3A, B**). The individual responses corrected with 11-1 concentration are shown in **Figures 3C, D**. Furthermore, we also analyzed EGFP-11 Nbs *via* AlphaFold2 software and determined interactions between EGFP-11-1 Nbs and CD147. It showed that EGFP-11-1 still had good affinity for CD147 (1.11 × 10^−8^ M) although EGFP protein fused into 11-1 Nbs (**Figures 3E, F**). It elucidated that Nb 11-1 could specifically bind CD147 or C78, and all data were analyzed using Octet Data Analysis Software or GraphPad Prism 5.

### 11-1 Specifically Recognized CD147 on Tumor Surfaces *In Vitro*

To detect whether anti-VHH-CD147 (11–1) bound specifically to CD147 proteins on cell surfaces, HeLa, SMMC-7721, and 293T [negative control (21)] cells were incubated with EGFP-11-1 or EGFP protein, respectively. It elucidated that EGFP-11-1 could significantly recognize the CD147 on the surfaces of HeLa and SMMC-7721 cells, but not 293T cells *via in vitro* laser scanning confocal microscopy imaging (**Figure 3G**). These data demonstrated that 11-1 Nbs could specifically recognize CD147 proteins on tumor cell surfaces.

### EGFP-11-1 Could Target Tumor *In Vivo* Assay

**Figure 4:**
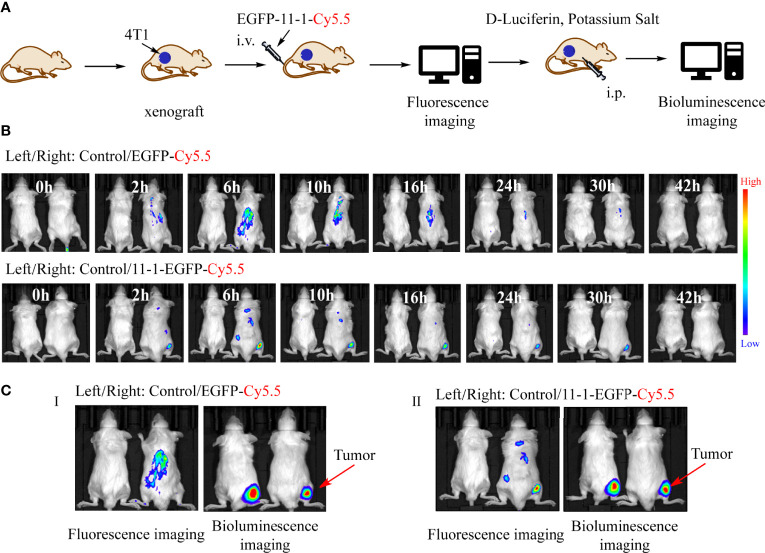
The tumor-targeting effects of anti-CD147 nanobody *in vivo* assays. **(A)** The scheme of tumor targeting of 11-1 nanobody. **(B)** The fluorescent images were observed in tumor at 0, 2, 6, 10, 16, 24, 30, and 42 h post-injection of PBS, EGFP-Cy5.5, and EGFP-11-1-Cy5.5, respectively. **(C)** The bioluminescence images were observed in tumor at 6 h post-injection of PBS, EGFP-Cy5.5, and EGFP-11-1-Cy5.5, respectively.

We used Cy5.5-NHS to label EGFP and EGFP-11-1 as described previously (51). Fluorescence images were obtained after the injection of EGFP-11-1-Cy5.5 and EGFP-Cy5.5 into Balb/c mice bearing 4T1 tumor cells in different time points. Among them, PBS injection was used as a blank control, and EGFP-Cy5.5 injection served as the negative control. A fluorescent signal was distinctly detected at the tumor regions after 2 h and became more intense over time, peaking at 6 h post-injection and then weakening gradually from 9 h post-injection until its complete disappearance at 42 h post-injection with EGFP-11-1-Cy5.5. The negative control showed no fluorescent signal at the tumor regions during the observation period (**Figures 4A, B**). Further bioluminescence imaging assays indicated that EGFP-11-1-Cy5.5 could target 4T1 breast cancer though intraperitoneal injection of D-Luciferin potassium salt (**Figure 4C**).

### DOX–11-1 Synthesis

**Figure 5:**
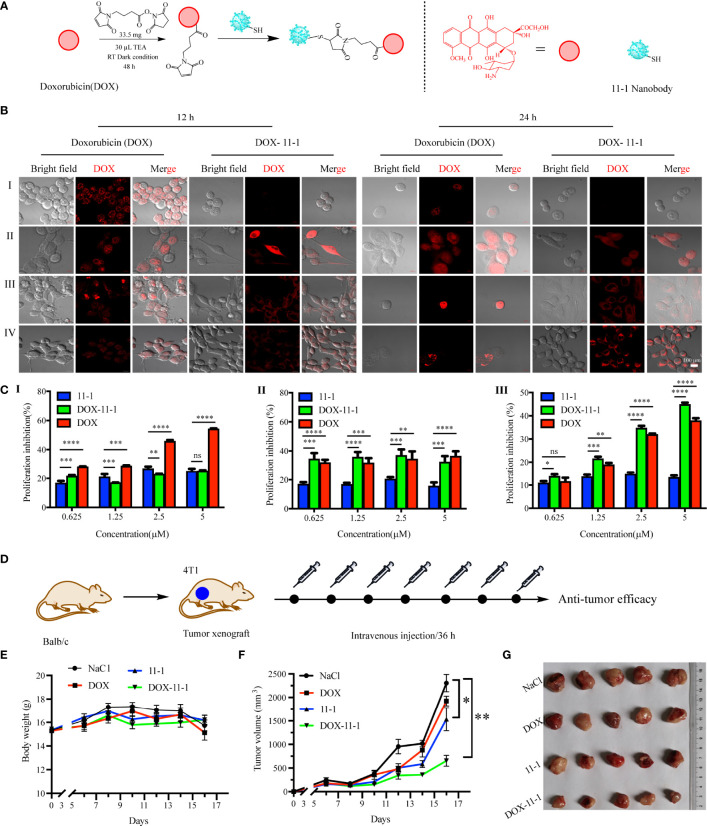
The antitumor efficacy of 11-1, free DOX, and DOX–11-1 *in vitro* and *in vivo* assays. **(A)** The scheme of synthesis DOX–11-1. **(B)** The distributions of free DOX and DOX–11-1 in different cell lines were observed by confocal laser scanning microscopy. (I) 293T, (II) U87, (III) HeLa, and (IV) 4T1 cells were treated with free DOX or DOX–11-1, and the fluorescence signal was observed by confocal laser scanning microscopy in indicated time points. Scale bar = 100 μm. **(C)** Antitumor effects of Nb 11-1, free DOX, and DOX–11-1 on different cells. Proliferation inhibition rates for 293T (I), U87 (II), and 4T1 (III) cells at 24 h after incubation with Nb 11-1, free DOX, and DOX–11-1, determined by MTT assay. **(D)** The scheme of DOX–11-1 antitumor efficacy. **(E)** The body weight change of mice bearing 4T1 xenografts *via* the treatment of 11-1, free DOX, DOX–11-1, and saline, respectively. **(F)** The tumor volume was significantly inhibited in DOX–11-1 compared with another group at 16 days. **(G)** The tumor photographs were shown at 16 days. Comparisons between two groups were calculated using one- or two-tailed Student’s *t*-tests, using GraphPad Prism software. Data are reported as mean ± SEM. Number of replicates (*n* = 5). *p < 0.05, **p < 0.01, ***p < 0.001, ****p < 0.0001, ns, no significance.

DOX–11-1 was obtained by chemical synthesis (**Figure 5A**). HPLC-MS confirmed that DOX-maleimide was highly pure, given the appearance of the major peak at 2.46 min. MS confirmed that the actual and theoretical molecular weights of DOX-maleimide were consistent (**Figure S5**). Free DOX-maleimide was removed using a desalting column to obtain the DOX–11-1 product (**Figure S6**).

### The Cellular Distribution of DOX–11-1

To compare the cellular distributions of DOX–11-1 and free DOX, CD147-positive cells were incubated with DOX–11-1 and DOX for 12 and 24 h, respectively. After 12 h of free DOX intervention, cell nuclei exhibited more quantitative red fluorescence; after incubation with DOX–11-1 for 12 h, a few red fluorescent signals were detected at the cytomembrane and cytoplasm (**Figure 5B**, left). After 24 h, significant red fluorescence could be seen in cell nuclei treated with free DOX and at the surfaces and cytoplasm of cells treated with DOX–11-1 (**Figure 5B**, right); the latter cells also showed a few fluorescent signals in nuclei, especially those of U87 and 4T1 cells (**Figures 5B**, II and IV). Negative control 293T cells showed no red fluorescence after the 12- and 24-h DOX–11-1 treatments, but significant fluorescence after the free DOX treatments (**Figures 5B**, I). The fluorescence intensity was positively correlated with time.

### The DOX–11-1 Could Inhibit Tumor Cell Proliferation

Fluorescence imaging assays proved that DOX–11-1 could enter the cell nucleus and cytoplasm of U87 and 4T1 cells, which prompts us to test whether DOX–11-1 could suppress U87 or 4T1 cell proliferation through DOX inducing DNA damage response. As shown in **Figure 5C**, 11-1 had little cytotoxicity effect on 293T, U87, and 4T1 cell lines. There were differences in the inhibition rate of U87 and 4T1 cells between the 11-1 group and DOX–11-1 or free DOX, and their inhibition rate reached approximately 40%–50% with significant treatment of DOX–11-1 or free DOX. Free DOX had a significant cytotoxicity effect on 293T, U87, and 4T1 cells at higher concentrations. However, little cytotoxicity was found in 293T cells treated with DOX–11-1 even at higher concentrations (5 μM). It indicated that the tumor cells are more sensitive to DOX–11-1 *in vitro*. Moreover, the safety concentration of DOX in 293T was considered to be below 2.5 μM in a subsequent *in vivo* study.

### DOX–11-1 Induced Cell Apoptosis

Apoptosis assays were further performed for 293T, U87, and 4T1 cells treated with different concentrations of Nb 11-1, DOX, and DOX–11-1. Apoptosis was detected by flow cytometry after treatment with 5 μM of these drugs (**Figures S7**). Minimal apoptosis of 293T cells was detected after the Nb 11-1 and DOX–11-1 interventions. Free DOX and DOX–11-1 caused the apoptosis of U87 and 4T1 cells, and free DOX additionally induced 293T cell apoptosis. The finding elucidated that DOX–11-1 could selectively induce CD147-positive cell apoptosis, which provides evidence for further research on antitumor mechanism.

### DOX–11-1 Inhibited 4T1 Tumor Growth in Balb/C Mice

4T1 tumor-bearing mice were treated with one time intravenous injections of DOX, 11-1, DOX–11-1, and saline, every 36 h. The antitumor scheme of DOX–11-1 is shown in **Figure 5D**. The mouse body weight had no obvious effect, and it implied that DOX–11-1 had good bio-safety (**Figure 5E**). Meanwhile, on day 16 after treatment, DOX–11-1 had a significant antitumor efficacy compared with another group (**Figures 5F, G**).

## Discussion

Accumulating lines of evidence demonstrate that CD147 plays pivotal roles in physiological and pathological processes. It was found that CD147 is widely distributed in the central nervous system, and its expression is consistent with the maturation of endothelial cells in the central nervous system, which serves as one of the markers of blood–brain barrier formation (52). Meanwhile, it is widely known as a potential biomarker and a promising target in cancer diagnosis and therapies. CD147 can lead to activation of ERK, which results in the degradation of Bim by the proteasome due to its own phosphorylation, and downregulation of Bim suppresses anoikis and promotes survival of cancer cells (53). Moreover, it has been reported that CD147-CAR (chimeric antigen receptor) can effectively induce human immune cells to target and kill various malignant HCC cells *in vitro* and *in vivo* (54). More importantly, CD147 is also a novel route for SARS-CoV-2 infection to host cells, which provides a crucial target for developing specific and effective drugs against COVID-19 (55, 56). Herein, a high-affinity anti-CD147-VHH Nb, coined as 11-1, was screened from a VHH phage antibody library that was derived from a healthy Bactrian camel immunized with the CD147 protein. The amino acid sequence of 11-1 has four cysteines, and cellular distribution of DOX–11-1 was *via* the cytoplasm rather than *via* the nucleus. Additionally, we used an online tool (prosite.expasy) to predict a protein’s disulfide bonds. These results elucidate that 11-1 (11-1-EGFP) has one disulfide bond. Of note, the CDR1 and CDR3 domains have one free Cys residue (data not shown). It has good targeting properties for HeLa, SMCC-7721, and 4T1, which highly express CD147 in *in vitro* or *in vivo* assays.

DOX, which serves as a first-line chemotherapeutic drug, is widely used for cancer therapies and fundamental scientific studies (57, 58). In this work, we determined that DOX was conjugated to 11-1 Nb *via* chemical modification, donated as DOX–11-1, and evaluated its antitumor efficacy in *in vitro* and *in vivo* assays. Results of *in vitro* assays demonstrated that there were significant differences between DOX and DOX–11-1 in cellular distributions. Among them, DOX–11-1 was detected in the membrane and cytoplasm of HeLa and 4T1 cells, which highly expressed CD147, but not in 293T cells with a low expression of CD147. We also validated that DOX–11-1 could induce more apoptosis in CD147 higher-expression cells. In *in vivo* assays, we chose 4T1 breast cancer as a model and found that 11-1 Nb could target the 4T1 tumor regions in Balb/c mice. Meanwhile, DOX–11-1 has excellent antitumor efficacy compared with other groups of 4T1-bearing Balb/c mice. However, there is an interesting result in which Nbs 11-1 had a slight antitumor efficacy in *in vivo* assays but not in *in vitro* assays. These results indicate that there may be some unknown mechanisms, including the tumor microenvironment, and immune responses involved in the *in vivo* antitumor effect of DOX–11-1 besides its tumor-targeting effect.

To date, more and more lines of evidence validate that the cancer cells that modulate the tumor microenvironment play important roles in the development, progression, and resistance to therapy in oncologic pathology (59). However, CD147 is closely related to pathological angiogenesis and inflammatory responses such as rheumatoid arthritis and atherosclerosis. Hence, anti-CD147/BSG- and CD147-related inhibitors that may regulate the immune system as novel antitumor drugs or antiangiogenic agents have been developed and used for the treatment of cancer (60). It indicates that CD147-targeted antibodies (11-1 or DOX–11-1) may be involved in regulating the tumor microenvironment and contributed to *in vivo* tumor inhibition effects. Definitely, the mechanisms responsible for this immunoregulative antitumor effects are incompletely understood. It will be of interest to explore these potential mechanisms in the future.

## Conclusion

In this study, a novel soluble anti-CD147-VHH Nb (11–1) was screened from a large synthetic phage display library containing specific VHH antibodies against the CD147 protein and obtained by prokaryotic expression. Furthermore, DOX–11-1 was successfully synthesized through the chemical coupling method, and it had potential antitumor activities not only in *in vitro* cells with a high expression of CD147 but also in *in vivo* experiments. In short, 11-1 is a promising Nb that will provide a novel strategy for the treatment of highly expressed CD147 tumors.

## Data Availability Statement

The original contributions presented in the study are included in the article/**Supplementary Material**, without undue reservation. Further inquiries can be directed to the corresponding authors.

## Ethics Statement

The animal study was reviewed and approved by the Institutional Authority for Laboratory Animal Care of Xuzhou Medical University, Xuzhou, China (accreditation number of the laboratory: 202007A229).

## Author Contributions

RL, YL, XZ, and XS conceived and designed the idea of the present work. PZ, YQ, and YX performed experiments and data analysis. RL wrote the initial manuscript drift and XZ revised it. All authors contributed to the article and approved the submitted version.

## Funding

This work was supported by Fundamental Research Funds for Hainan University [KYQD(ZR)-21109], the National Natural Science Foundation of China (No. 81800916), the Natural Science Foundation of the Jiangsu Higher Education Institutions of China (19KJA560002), the Xuzhou Key R&D Program (Social Development) (No. KC20177), and the Xiaogan Natural Science Program Project. XGKJ2021-010036.

## Conflict of Interest

The authors declare that the research was conducted in the absence of any commercial or financial relationships that could be construed as a potential conflict of interest.

## Publisher’s Note

All claims expressed in this article are solely those of the authors and do not necessarily represent those of their affiliated organizations, or those of the publisher, the editors and the reviewers. Any product that may be evaluated in this article, or claim that may be made by its manufacturer, is not guaranteed or endorsed by the publisher.

## Abbreviations

CD147, extracellular matrix metalloproteinase inducer; C78, C-terminal ectodomain protein of CD147; VHH, the variable domain of heavy chain of heavy-chain antibody; ELISA, enzyme-linked immunosorbent assay; BLI, biolayer interferometry; IgSF, immunoglobulin superfamily; MCTs, monocarboxylate transporters; HCAb, heavy-chain antibody; PBS, phosphate buffered saline; BSA, bovine serum albumin; IPTG, isopropyl-β-d-thiogalactoside; SDS-PAGE, sodium dodecyl sulfate polyacrylamide gel electrophoresis; Nb, nanobody; PVDF, polyvinylidene fluoride; CFU, colony-forming units; pI, isoelectric point; ϵ, extinction coefficient; II, instability index; AI, aliphatic index.
