# Supplementary material for: Generation of a High-Affinity Nanobody Against CD147 for Tumor Targeting and Therapeutic Efficacy Through Conjugating Doxorubicin
Source: Front Immunol. 2022 May 4;13:852700. doi: 10.3389/fimmu.2022.852700 (PMC9114487; doi:10.3389/fimmu.2022.852700)
Supplement: Supplementary file 1 [file DataSheet_1.docx]

Supplementary Material

## Supplementary Figures


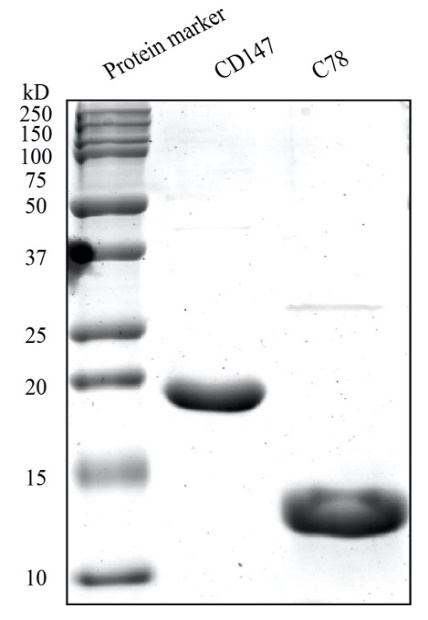


**Supplementary Figure 1.** Analysis of purified CD147 and C78 (C-CD147) proteins by SDS-PAGE.


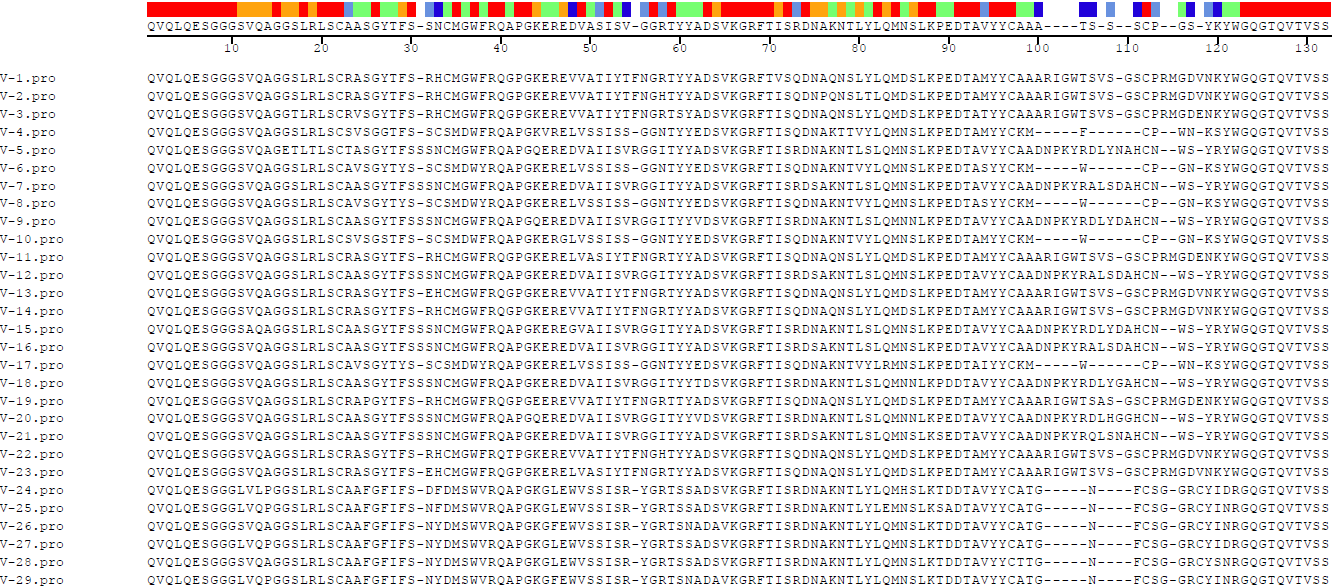


**Supplementary Figure 2.** The sequences of the 29 positive clones for α-CD147 nanobodies.


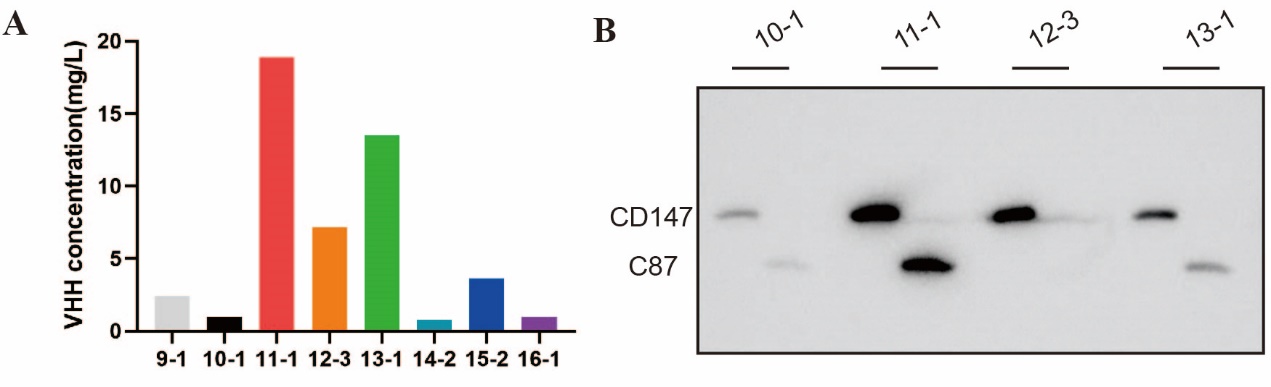


**Supplementary Figure 3.** The expression yield and specificities of α-CD147 nanobodies. (A) The expression yield of α-CD147 nanobodies. (B) The specificities of α-CD147 nanobodies (10-1, 11-1, 12-3, 13-1).


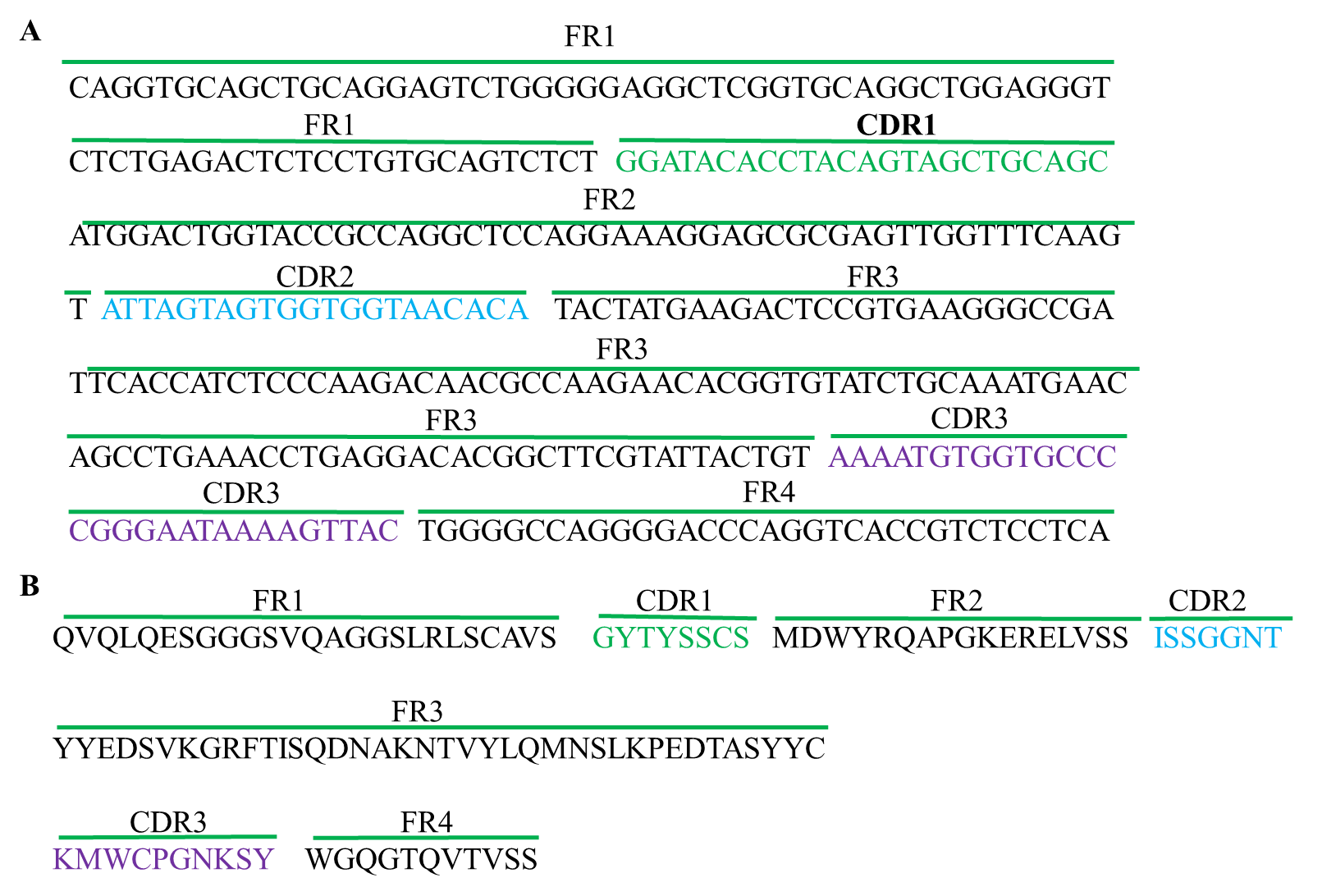


**Supplementary Figure 4.** 11-1 nanobody sequence analysis. (A) The nucleo-base sequence of 11-1. (B) The amino acid sequence of 11-1.


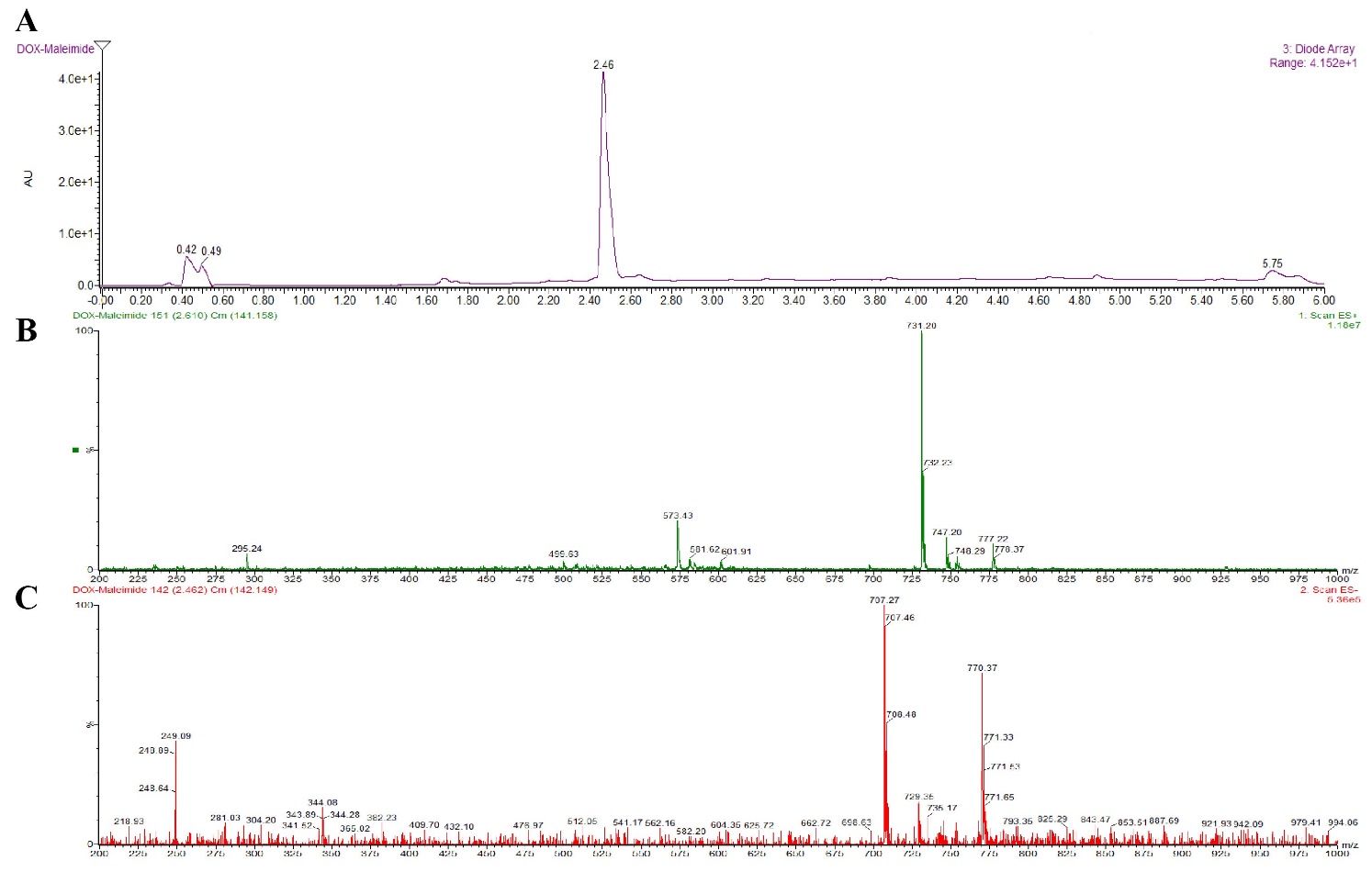


**Supplementary Figure 5.** The determination of DOX- maleimide. (A) DOX-maleimide separation, observed by LC. (B-C) Mass of DOX-maleimide (C) M+Na^¬+^ (D) M-H^¬-^. calcd for C_35_H_37_N_2_O_14_^+^ 707.67，found 707.27.


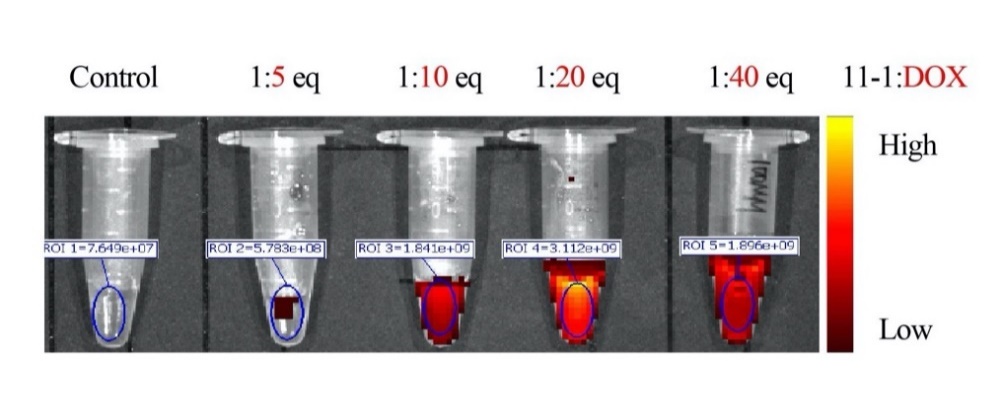


**Supplementary Figure 6.** The conjugation of 11-1 with doxorubicin. 11-1 reaction than with doxorubicin were 0 (Control), 1:5 eq, 1:10 eq, 1:20 eq, 1:40 eq at 25 ℃, respectively.


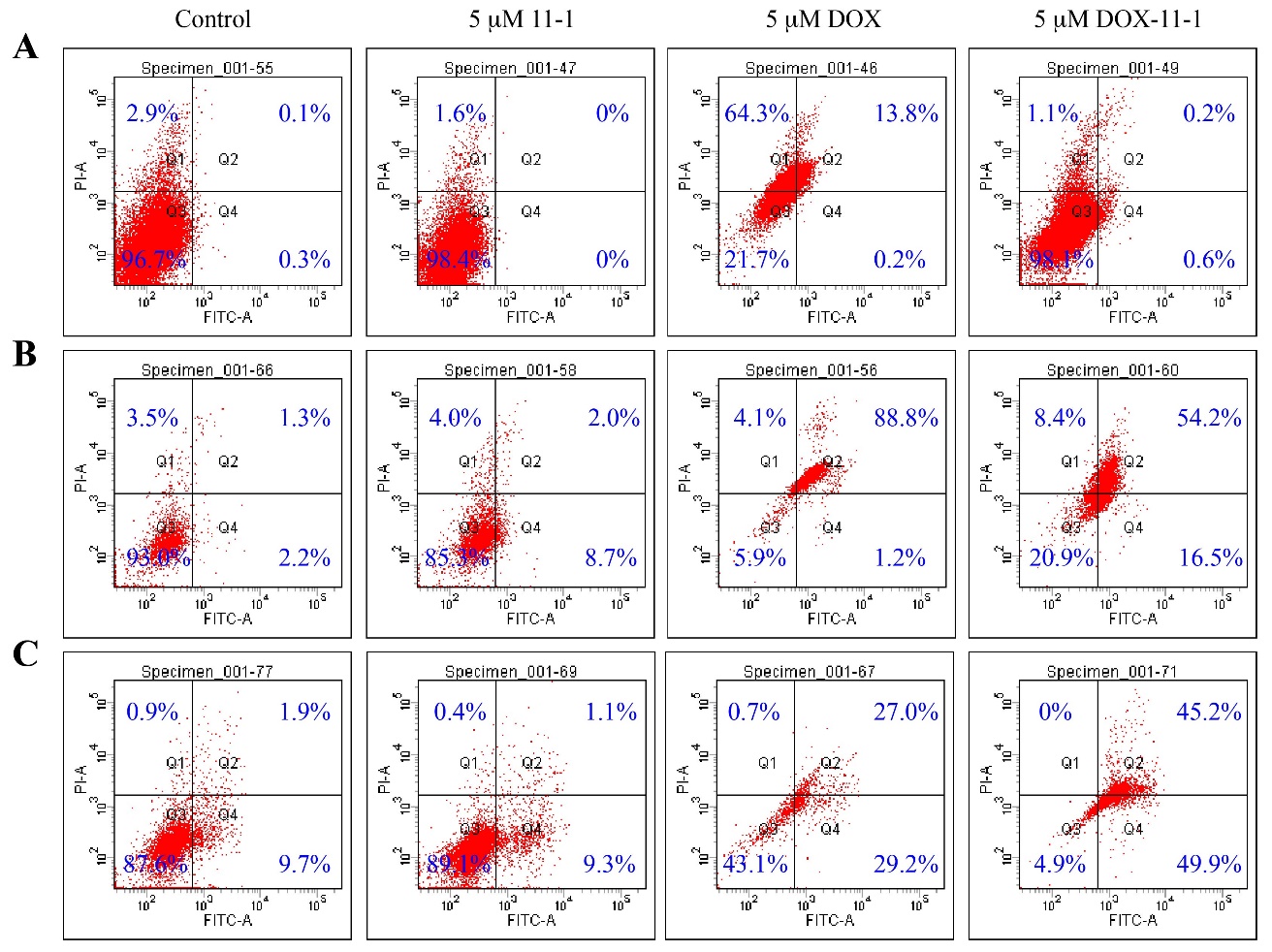


**Supplementary Figure 7.** Apoptotic and necrotic cell detection by annexin V-FITC/PI assay. The cells were incubated with 5 μM Nb 11-1, DOX, and DOX–11-1 for 24 h. (A) 293T cells, (B) U87 cells, and (C) 4T1 cells.
